# Ruptured distal anterior cerebral artery aneurysm presenting with acute subdural hematoma without subarachnoid hemorrhage

**DOI:** 10.1093/jscr/rjae843

**Published:** 2025-01-09

**Authors:** Michio Yamazaki, Taisei Aoki, Fumihiro Matano, Yasuo Murai

**Affiliations:** Department of Neurosurgery, Tama Nagayama Hospital, Tokyo, Japan; Department of Neurological Surgery, Nippon Medical School Hospital, Tokyo, Japan; Department of Neurological Surgery, Nippon Medical School Hospital, Tokyo, Japan; Department of Neurological Surgery, Nippon Medical School Hospital, Tokyo, Japan

**Keywords:** anterior cerebral artery, cerebral aneurysm, subarachnoid hemorrhage, subdural hematoma

## Abstract

We report a case of distal anterior cerebral artery (DACA) aneurysm presenting with subdural hematoma (SDH) without subarachnoid hemorrhage (SAH). A patient in his fifties presented with headache. Fluid-attenuated inversion recovery magnetic resonance imaging revealed SDH in the interhemispheric fissure and left frontotemporal region. SAH was not detected. Computed tomographic angiography revealed aneurysms in the left internal carotid artery (ICA) and DACA. The patient underwent frontotemporal craniotomy, which confirmed an unruptured ICA aneurysm, followed by bilateral frontal craniotomy for clipping of DACA aneurysm. The left DACA aneurysm was identified as the source of the SDH. Intraoperative findings showed adhesion between the aneurysm body and falx cerebri, explaining the SDH formation. Literature review identified only six reported cases of SDH without SAH due to DACA aneurysm. Evidence suggests that DACA aneurysms have a relatively higher propensity to cause SDH without SAH, likely due to the anatomical characteristics of the parent vessel and aneurysm projection.

## Introduction

Aneurysmal rupture accounts for 0.5%–6.9% [1–3] of non-traumatic acute subdural hematomas (SDH). While ⁓80% [4–6] of these cases involve internal carotid artery (ICA) or anterior cerebral artery aneurysms, distal anterior cerebral artery (DACA) aneurysms account for ⁓11.2%–20.1% [4, 5, 7]. Pure SDH without subarachnoid hemorrhage (SAH) or intracerebral hemorrhage from ruptured aneurysms are rare, with only 34 reported [4, 5, 7] cases in the literature, including our case. Of these, only seven cases [5, 8–12], including ours, were caused by DACA aneurysm rupture. This case report discusses the clinical characteristics, diagnostic considerations, and important therapeutic aspects of pure SDH caused by ruptured DACA aneurysm, a relatively rare clinical presentation. Through our experience and literature review, we highlight the unique features of this condition and provide practical insights for its diagnosis and management.

## Case report

**Figure 1:**
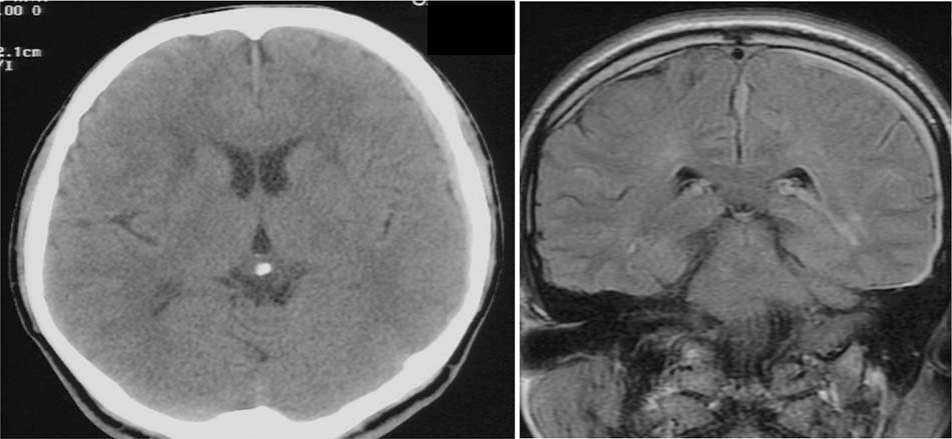
CT (left) axial image of the brain and MRI (right) fluid-attenuated inversion recovery coronal image from the referring hospital. SDH is visible on both images, without evidence of SAH. SDH is also visible in the interhemispheric fissure on MRI.

**Figure 2:**
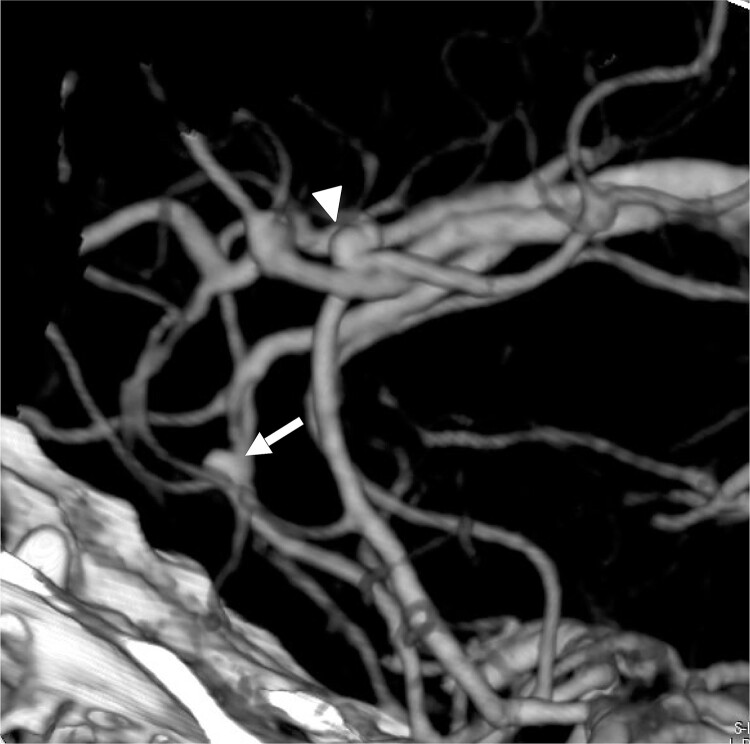
Lateral view (left to right) of three dimensional CT angiography taken at our clinic, showing cerebral aneurysm in the ACA of right A2A3 (white arrow) and left A3A4 (white arrow head).

**Figure 3:**
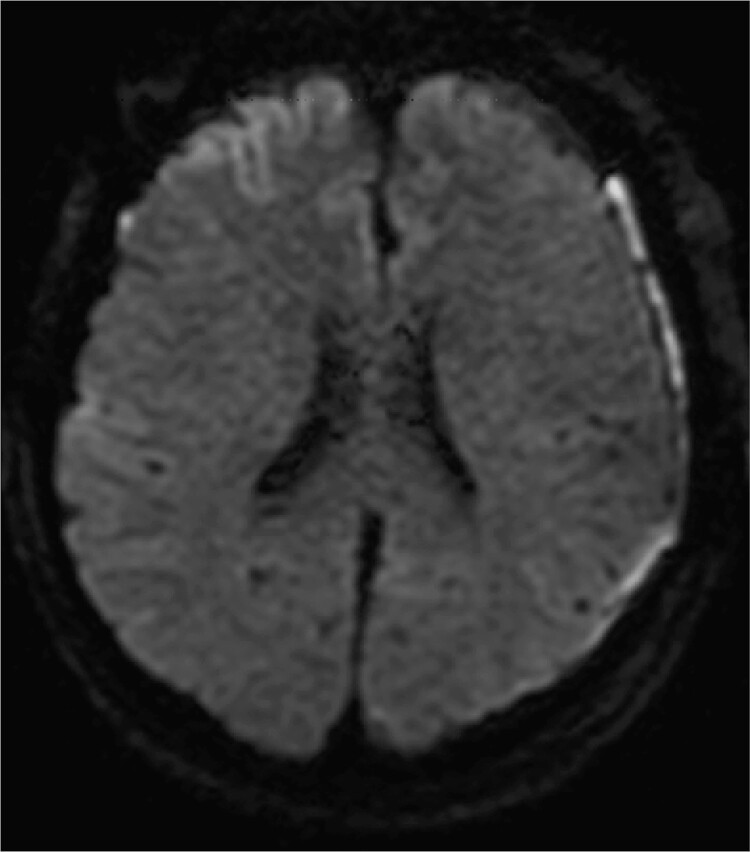
Diffusion-weighted images on postoperative day 3 are shown. No obvious ischemic changes are seen.

A man in his fifties presented with sudden severe headache that showed no improvement. Initial CT at another hospital revealed SDH, prompting referral to our institution. Head CT demonstrated left SDH including the interhemispheric fissure (Fig. 1). 3D-CTA performed at the referring hospital revealed ⁓5-mm saccular aneurysms at the left ICA and right basilar-superior cerebellar artery junctions, along with left A3-A4, and right A2-A3 aneurysms (Fig. 2). Preoperative FLAIR imaging showed no evidence of SAH (Fig. 3). As the initial 3D-CTA inadequately captured the distal portions of the ACA, we performed repeat imaging upon admission, which revealed an additional left A3A4 DACA aneurysm. However, due to the DACA aneurysm being smaller than the ICA aneurysm, definitive determination of the rupture source was not possible.

Given the uncertainty regarding the rupture site, we first performed left frontotemporal craniotomy, which confirmed an unruptured ICA aneurysm. Subsequently, bilateral frontal craniotomy was performed with careful evacuation of the SDH. The callosomarginal artery was readily identified in a relatively shallow subarachnoid space during interhemispheric fissure dissection. No SAH was observed. The aneurysm body demonstrated strong adhesion to the left side of the falx cerebri, with white thrombus continuous with the SDH, identifying it as the rupture site. Both the left ICA and DACA aneurysms were successfully clipped. Postoperative diffusion-weighted imaging on day 3 showed no frontal cortical ischemic changes (Fig. 3). DACA flow without vasospasms was confirmed by cerebral angiography on day 8. The patient was discharged without neurological deficits.

## Discussion

We report a case of DACA aneurysm presenting with acute SDH without SAH, of which only six [5, 8–12] cases have been previously documented in English literature. Due to the presence of multiple aneurysms in our case, including an ipsilateral ICA aneurysm, preoperative identification of the rupture site was challenging. However, by approaching the surgery with consideration that the SDH was caused by aneurysmal rupture, we were able to manage the case appropriately, resulting in a favorable outcome without neurological deficits.

Several recent reviews [4, 5, 7] have discussed aneurysm-related SDH. English literature review revealed 56 [4, 7] cases of pure SDH without SAH or intracerebral hemorrhage. According to Gotan *et al.* [5], the distribution of aneurysms causing SDH was: ICA (50.9%), middle cerebral artery system (20%), and anterior communicating/anterior cerebral artery system (29.1%). Another literature review [7] of 36 pure SDH cases showed a similar distribution: ICA (52.9%, 18 cases), middle cerebral artery system (26.5%, 9 cases), anterior cerebral artery system (14.7%, 5 cases), and anterior communicating artery system (5.9%, 2 cases). According to Lehecka *et al.'s* study [13], among 3005 cases with 4253 cerebral aneurysms, 174 (4.1%) were DACA aneurysms. Similarly, Lee *et al.* [3] reported that out of 3577 cases of cerebral aneurysms, 126 cases (3.5%) were identified as DACA aneurysms. Agarwal *et al.* [14] found incidental aneurysms in 1.8% (39/2195) of 3D-CTAs, with 5.1% (two cases) being anterior cerebral artery aneurysms excluding anterior communicating artery aneurysms. UCAS data [15] shows distribution of unruptured aneurysms as: anterior communicating artery (15.5%), middle cerebral artery (36%), ICA (34%), and DACA (<5%). Globally, DACA aneurysms constitute ⁓5% of all cerebral aneurysms. However, they account for 23.6% of aneurysmal ASDH cases without SAH, suggesting a relatively higher tendency for this presentation.

Our literature review identified six previously reported cases of DACA aneurysms at the A2 segment presenting with SDH without SAH in English-language publications. Table 1 presents the clinical courses of seven cases [5, 8–12], including our case. The analysis revealed no gender distribution (three males, four females). Five cases involved left-sided lesions, while one case presented with an azygos ACA aneurysm with right A1 dominance. Five patients exhibited severe disturbance of consciousness upon admission. Regarding treatment approaches, three cases underwent simultaneous hematoma evacuation and aneurysm clipping in a single-stage procedure. In one case [9], hematoma evacuation was performed first, followed by staged clipping after aneurysm confirmation. There was one reported [10] case where the aneurysm was not identified preoperatively, and arterial bleeding encountered during hematoma evacuation was managed solely with bipolar coagulation. Additionally, one case report [5] described initial hematoma evacuation followed by staged endovascular treatment. Gotan *et al*. [5] reported a case where they initially performed hematoma evacuation through a frontotemporal craniotomy, followed by endovascular coiling of the aneurysm. They [5] noted that intraoperative rupture occurred during coiling, resulting in contralateral SDH, which necessitated additional coiling. Unlike the treatment of ruptured aneurysms presenting with SAH, there is no evidence regarding the therapeutic efficacy and prognosis of endovascular treatment for ruptured aneurysms presenting with acute SDH. Further accumulation of cases is needed. For cases presenting solely with SDH, treatment strategies should be considered in light of the fact that cerebral vasospasm associated with SAH does not occur. Regarding prognosis, the absence of SAH meant that no cases were affected by vasospasm [5, 6, 11]. Our case also progressed without postoperative vasospasm. Based on the clinical courses of both our case and previously reported cases, we conclude that appropriate initial diagnosis and preparation for adequate craniotomy that enables management of potential intraoperative rupture can lead to favorable outcomes.

**Table 1:** Characteristics and treatment outcomes of seven cases.

Author	Year	Age	Sex	Side	Location	WFNS grade on admission	Treatment	Treatment complication	GOS
Watanabe	1991	50s	M	L	A2A3	5	None	Intraprocedural rupture	6
Hashizume	1992	60s	M	R(Azygos)	Azygos	5	C & H	None	0
Nowak	1995	50s	F	R	pericallosal	5	H	None	6
Weil	2010	50s	F	L	A2A3	5	H & C (two stage)	None	unknown
Song	2016	40s	F	L	A3/A4	1	C & H	None	0
Gotan	2023	20s	F	L	A2A3	5	H & E (two stage)	Intraprocedural rupture. Contralateral SDH	4
Authors	2024	50s	M	L	A3/A4	1	C & H	None	0

GOS: Glasgow outcome scale; C: clipping of aneurysm; E: endovascular treatment; H: hematoma evacuation only; O: observation; F: female; M: Male; R: right; L: left; WFNS: World Federation of Neurosurgical Societies.
